# Depressive symptoms and healthcare utilization among older adults in China: A cross-sectional examination of the national CHARLS data guided by Andersen behavioral model

**DOI:** 10.1371/journal.pone.0337835

**Published:** 2025-12-04

**Authors:** Yue Wu, Teresa Wills, Aine O’Donovan, Caroline Kilty, Jin Liu, Jian Huang, Qingyue Wang, Nicola Cornally

**Affiliations:** 1 School of Nursing and Midwifery, University College Cork, Cork, Ireland; 2 Department of Nursing, The First Affiliated Hospital with Nanjing Medical University, Nanjing, China,; 3 Department of Science and Technology, The First Affiliated Hospital with Nanjing Medical University, Nanjing, China; 4 School of Mathematical Sciences, University College Cork, Cork, Ireland; Federal University of Paraiba, BRAZIL

## Abstract

**Background:**

With global population ageing, the mental health of older adults is a significant public health concern. Depression and depressive symptoms are increasingly prevalent and have been shown to influence healthcare utilization, but the mechanisms underlying these effects operate remain underexplored, particularly among older adults in China. Building on this context, this study draws on the Andersen behavioral model to explore the associations between depressive symptoms and healthcare utilization among older adults in China.

**Methods:**

Using data from the 2018 China Health and Retirement Longitudinal Study, a nationally representative sample of 7,777 individuals aged 60 and above was analyzed. A Bayesian Generalized Structural Equation Model was constructed within the framework of the Andersen behavioral model to estimate both the direct and indirect associations between depressive symptoms and inpatient healthcare utilization.

**Results:**

Results showed a direct association between depressive symptoms and inpatient healthcare utilization (0.16, 95% HDI: 0.02, 0.29), accounting for a small portion of the total association. The remaining 93.55% operated through indirect pathways, with health status, chronic diseases, satisfaction with health, activities of daily living limitations, disability, and alcohol use identified as key mediators. The model showed strong convergence and estimation stability.

**Conclusions:**

These findings highlight the importance of distinguishing between direct and indirect associations when evaluating the relationship between depressive symptoms and healthcare utilization. Such differentiation helps to clarify the underlying mechanisms and provides an empirical basis for developing more targeted interventions to improve healthcare service use among Chinese older adults with depressive symptoms.

## 1. Introduction

China, as the country with the largest older population, has 297 million individuals aged 60 and above, representing 21.1% of its total population, with 217 million aged 65 and above, comprising 15.4% [1]. The United Nations (UN) defines a society as aged when over 14% of the population is aged 65 and above, a threshold that China has already surpassed [2]. With the increase in population ageing, the burden of mental health issues among older people has become increasingly prominent [3]. This has prompted global and national policy to address the mental health challenges posed by an ageing population [4].

Against this backdrop, the UN declared 2021–2030 as the ‘Decade of Healthy Ageing’, emphasizing not only physical health but also mental well-being [5]. Within this global framework, the National Health Commission of China also released the ‘14th Five-Year Plan for Healthy Ageing’, designating mental health among older persons as a key focus [6]. These policies highlight the importance of promoting mental health and optimizing relevant services as central to addressing the challenges posed by an ageing population.

Depression is one of the most common mental health issues experienced by older adults, with a recent meta-analysis reporting a global prevalence of 35.1% [7]. Wu et al. [8] conducted an integrative review of 65 studies published between 2014–2023 and reported that the prevalence of depression and depressive symptoms among older adults in China ranges from 3.78% to 84.3%. This wide variation was attributed to assessment methods, sample characteristics, and regional differences [8]. Individuals with depression frequently utilize general healthcare services due to physical discomfort caused by depressive symptoms, but these somatic complaints are frequently not recognized as being related to depression [9]. However, limited awareness of mental health conditions, including depression, frequently results in reduced access to and underutilization of specialized mental health services [10]. This imbalance in accessing the right care at the right time exacerbates the strain on healthcare resources [11] and can lead to delayed treatment resulting in poorer patient outcomes [12]. In this context of limited healthcare resources, the strategic allocation and optimization of service use have been identified as critical areas of focus [13].

Evidence from China is limited, international studies indicate a positive association between depressive symptoms and both primary and inpatient service use [14]. However, national data reveal extremely low treatment coverage for depressive disorders [15], suggesting that this relationship may operate largely through indirect mechanisms. Meanwhile, the rapid growth of inpatient care relative to outpatient care [16] underscores mounting pressure on hospital resources and the need to better understand inpatient utilization patterns.

However, there is a lack of theory-driven research that examines how depressive symptoms influence healthcare utilization, particularly with regard to potential mediating processes, within well-established conceptual frameworks.

Guided by the Andersen behavioral model [17], this study employs Bayesian Generalized Structural Equation Model (BGSEM) [18] to examine the associations between depressive symptoms and inpatient healthcare utilization among older adults and determine the underlining mediating mechanism. The Andersen behavioral model conceptualizes healthcare utilization as determined by three domains: predisposing factors, enabling factors, and need factors. Two systematic reviews [19,20] have summarized how these domains are commonly operationalized and provide direct guidance for the classification and selection of variables in this study. The analysis examined the overall association between depressive symptoms and inpatient healthcare utilization and further explored whether this relationship operated through specific mediating variables, including, pain [21], smoking [22], alcohol use [23], disability [24], health status [25], satisfaction with health [26], limitations in activities of daily living (ADL) [27], and chronic diseases [28].

Although the terms depression and depressive symptoms are often used interchangeably in literature, this study adopts the latter to refer specifically to symptom-based self-report rather than clinical diagnosis [29]. This decision is grounded in both the nature of the available data and the practical context in which older adults typically seek care for physical discomfort rather than explicitly for depression.

## 2. Methods

### 2.1 Data source

This cross-sectional study used data from the 2018 wave of the China Health and Retirement Longitudinal Study (CHARLS), a nationally representative survey of Chinese residents aged 45 years and older. The survey adopted a stratified, multi-stage, probability proportional to size (PPS) random sampling design across 28 provinces, covering 150 counties or districts and 450 villages or urban communities. In the full sample, 8.1% of interviews were completed by a proxy respondent for participants who were physically or cognitively impaired, hospitalized, unreachable during fieldwork, or unwilling to participate in person. A total of 19,816 individuals were interviewed in 2018. The dataset is publicly available at http://opendata.pku.edu.cn/. The data used in this study were accessed on December 13, 2024. The dataset was fully de-identified, and the authors had no access to any personally identifiable information. Details of the study design and objectives have been reported by Zhao et al. [30].

All participants or their legal representatives provided written informed consent to participate in the baseline and follow-up surveys. The CHARLS study was approved by the Biomedical Ethics Review Committee of Peking University (IRB00001052−11015). This study was conducted in accordance with the Strengthening the Reporting of Observational Studies in Epidemiology (STROBE) guidelines for cross-sectional studies [31]. In addition, the Bayesian data analysis was informed by the ROBUST (Reporting Of Bayes Used in clinical Studies) framework [32] to promote transparency and reproducibility in the modelling approach. For the secondary analysis conducted as part of this study, an ethical review was completed by the Social Research Ethics Committee of University College Cork (Log number: 2024−295).

All data processing and statistical analyses were conducted using R Studio [33] and R [34].

### 2.2 Participants

**Fig 1:**
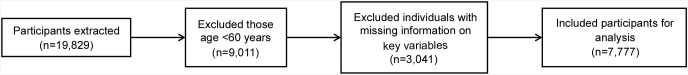
The flow chart of subject selection.

The study population comprised older adults aged 60 years and above, selected from the 2018 CHARLS dataset. During data cleaning, individuals with incomplete data for ID (Identification Number), survey weights, and key study variables (depressive symptoms, and inpatient healthcare utilization) were excluded, resulting in the removal of 3,041 cases. The final analytical sample consisted of 7,777 participants. The flow chart of subject selection is presented in Fig 1.

### 2.3 Variable selection

The selection of variables in this study was grounded in the Andersen behavioral model, which encompasses predisposing, enabling, and need factors and is widely used to examine factors influencing healthcare utilization.

#### 2.3.1 Constructing the variable framework.

This study referenced two systematic reviews [19,20] to identify commonly used variables in healthcare utilization research and refine variable selection within the Andersen behavioral model framework. Guided by this framework and the supporting literature, we mapped relevant CHARLS questionnaire items to the identified candidate variables, which formed the final set used for analysis (Table 1). Details of variable selection and theoretical rationales based on the Andersen behavioral model are provided in S1 File.

**Table 1:** Summary of variables based on Andersen behavioral model.

Details or comparator	
**Predisposing factors**	
Gender	Female/Male
Age	60-69/70-79/ ≥ 80
Marital status	No/Yes
Ethnicity	Non-Han/Han
Residence	Urban/Rural
Religious belief	No/Yes
Work status	No/Yes
Smoking	No/Yes
Alcohol use	No/Yes
**Enabling factors**	
Education	Illiterate/Primary/Secondary/Higher education
Pension	No/Yes
Satisfaction with healthcare services	Dissatisfied/Neutral/Satisfied
Physical examination	No/Yes
Health insurance	No/Yes
**Need factors**	
Pain	No/Yes
Chronic disease	No/Single/Multiple
Disability	No/Yes
ADL	No Limitation/ Mildly Limited/Moderately Limited/Severely Limited
Health status	Poor/Neutral/Good
Satisfaction with health	Dissatisfied/Neutral/Satisfied

#### 2.3.2 Identifying mediating variables.

Building on the construction of variables, this study further identified potential mediators between depressive symptoms and inpatient healthcare utilization. The initial variable framework was developed based on the Andersen behavioral model, emphasizing variables that are theoretically linked to healthcare utilization. Subsequently, guided by the mediation criteria proposed by Baron and Kenny framework [35], we incorporated variables that demonstrated directional associations with depressive symptoms in previous studies as mediators in the model.

Longitudinal research has shown that depressive symptoms predict the subsequent onset of low back pain [21], declines ADL functioning [27], and the occurrence or progression of chronic diseases [28], thereby offering temporal-sequence support for these pathways. In addition, several cross-sectional studies have modelled depressive symptoms as explanatory variables and reported directional associations with alcohol use [23], health status [25], and satisfaction with health [26]. The smoking pathway is further supported by neurobiological evidence involving alterations in the reward system [22]. Moreover, depression is widely recognized as a major contributor to disability burden [24], and macro-level epidemiological trend analyses have provided population-level support for its relationship with disability. Based on this evidence, eight mediators were included in the model: pain [21], smoking [22], alcohol use [23], disability [24], health status [25], satisfaction with health [26], ADL [27], and chronic diseases [28].

#### 2.3.3 Determining control variables.

**Fig 2:**
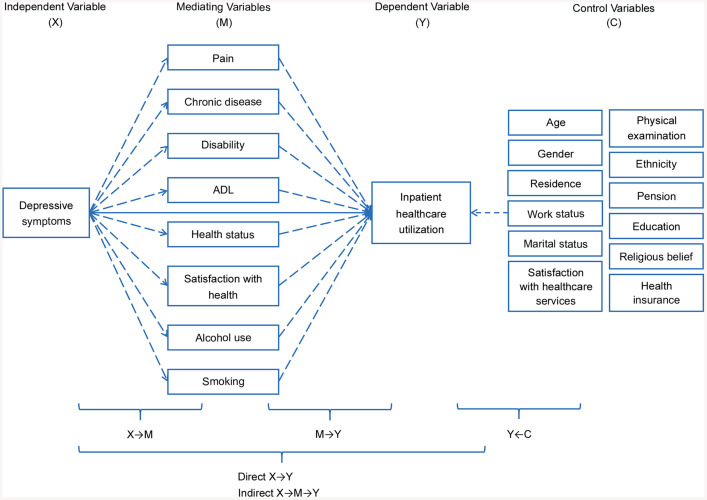
Conceptual framework diagram.

Other variables within the Andersen behavioral model that are known to influence healthcare utilization, particularly inpatient services, but are not directly affected by depressive symptoms, were classified as control variables. This approach follows Spector & Brannick’ s recommendation that control variables should be theoretically justified and associated with the outcome of interest [36]. These include gender, age, education, marital status, work, pension, health insurance, residence, and physical examination, ensuring that potential confounders are accounted for in the analysis, as shown in the conceptual framework (Fig 2).

This variable selection process integrates theoretical foundations, empirical evidence, and data feasibility, including the presence of variables in the CHARLS dataset, consistency in measurement, and overall usability, to ensure the robustness and applicability of the analytical model.

#### 2.3.4 Independent variable: depressive symptoms.

Depressive symptoms were assessed using the 10-item Centre for Epidemiologic Studies Depression Scale (CES-D-10) [37]. Each item was rated on a 4-point Likert scale (0–3), yielding a total score ranging from 0 to 30. A cutoff score of ≥10 was used to indicate the presence of depressive symptoms, while scores <10 were categorized as an absence of depressive symptoms.

#### 2.3.5 Dependent variable: inpatient healthcare utilization.

In the CHARLS dataset, inpatient care encompasses a range of institutional settings, including general hospital, specialized hospital, Chinese medical hospital, community healthcare center, township hospital, health care post, nursing home. This study adopts a systems-level perspective on healthcare delivery and employs a broad conceptualization of inpatient care to explore the potential pathways linking depressive symptoms to hospitalization.

Detailed definitions, measurement procedures, and coding of other variables are provided in S2 File.

### 2.4 The processing of missing data

Little’s χ² test indicated that missing data were not completely at random (MCAR, χ² = 1379, df = 42, p < 0.001) [38]. To mitigate estimation bias while preserving statistical power, this study employed the Multiple Imputation by Chained Equations (MICE) approach to handle missing values in key variables [39].

To assess the validity of the imputation, distributional comparisons were conducted between observed and imputed data using graphical methods. Histograms indicated that the imputed values closely aligned with the original data distribution, confirming that the imputation procedure successfully generated plausible estimates. This principled imputation strategy enhances the reliability of subsequent model estimations and reduces potential bias introduced by missing data. Detailed information on missing variables is provided in S1 Table.

### 2.5 Structural equation model

This study employed a Bayesian Generalized Structural Equation Model [18] within the framework of the Andersen behavioral model to estimate both the direct and indirect associations between depressive symptoms and inpatient healthcare utilization. BGSEM was selected for its capacity to model complex mediation pathways and to quantify effect uncertainty through posterior distributions. The model incorporates normalized survey weights to ensure sample representativeness.

To assess multicollinearity prior to model estimation, adjusted generalized variance inflation factors (aGVIFs) were computed for all explanatory variables, including depressive symptoms, mediators and control variables [40].

To evaluate different model specifications, two modelling approaches were considered:

1) Comprehensive Model: This model was specified based on the Andersen behavioral model and included all mediators and control variables considered theoretically relevant. It represents the full structural specification to examine the pathways linking depressive symptoms to inpatient healthcare utilization.
2) Refined Model: This model was specified as an alternative formulation using a subset of variables drawn from the comprehensive model. Variable exclusion was guided by posterior summaries, particularly 95% HDIs that include zero, and posterior mean estimates indicating limited effect sizes. The model structure was defined to remain consistent with the theoretical framework.

#### 2.5.1 Model specification.

In the BGSEM framework, inpatient healthcare utilization (Y) is modelled as a function of depressive symptoms (X), mediators ($M_{k}$), and control variables ($C_{j}$). The model consists of the following equations:

1) Outcome model

The probability of inpatient healthcare utilization ($Y_{i}$) is estimated using a Bernoulli-logit function:


$$\text{log}\left(\frac{P\left(Y_{i}=\text{1}\right)}{\text{1}-P\left(Y_{i}=\text{1}\right)}\right)=\alpha_{Y}+\beta_{X}X_{i}+\sum_{k}\beta_{k}M_{ki}+\sum_{j}\beta_{j}C_{ji}$$

Where:

$Y_{i}$= = Inpatient healthcare utilization

$\alpha_{Y}$ = Intercept

$\beta_{X}X_{i}$ = Direct effect of depressive symptoms $X_{i}$ on inpatient healthcare utilization$\;Y_{i}$

$\sum_{k}\beta_{k}M_{ki}$ = Effects from mediators $M_{ki}$

$\sum_{j}\beta_{j}C_{ji}$ = Effects from control variables $C_{ji}$

2)Mediation models

Each mediator ($M_{k}$) is modelled as a function of depressive symptoms (X) using the following specifications:

Binary mediators are modelled using a Bernoulli-logit function:


$$\text{log}\left(\frac{P\left(M_{ki}=\text{1}\right)}{\text{1}-P\left(M_{ki}=\text{1}\right)}\right)=\alpha_{k}+\gamma_{k}X_{i}$$

$M_{ki}$ = Binary mediators

$\alpha_{k}$ = Intercept

$\gamma_{k}$ = Effect of depressive symptoms $X_{i}$ on the binary mediator $M_{ki}$

Ordinal mediators modelled using a Cumulative-logit function:


$$\text{log}\left(\frac{P\left(M_{ki}\leq m\right)}{P\left(M_{ki}>m\right)}\right)=\alpha_{m}+\gamma_{k}X_{i}$$

Where:

$M_{ki}$ = Ordinal mediator

m = Threshold for ordinal categories

$\alpha_{m}$ = Threshold parameters for category m

$\gamma_{k}$ = Effect of depressive symptoms $X_{i}\;$on ordinal mediator $M_{ki}$

#### 2.5.2 Model estimation and computational settings.

All models were estimated using Markov Chain Monte Carlo (MCMC) with the No-U-Turn Sampler (NUTS), a variant of Hamiltonian Monte Carlo (HMC), implemented via CmdStan in the brms package. NUTS adaptively optimizes step sizes, ensuring efficient posterior exploration and enhancing computational stability [41]. Models were run with 4 parallel chains and 10,000 iterations per chain, including 5,000 warm-up iterations, ensuring sufficient sampling for reliable posterior inference. Weakly informative priors were applied to improve numerical stability and provide regularization without unduly influencing the results [41]. A Cauchy (0, 2.5) prior was specified for all regression coefficients. For intercepts and threshold parameters in ordinal models, the default standard priors in the brms package (Student-t (3, 0, 2.5)) were applied.

Model convergence was evaluated using the Gelman-Rubin statistic (R-hat) to ensure proper chain mixing, with values close to 1.00 indicating convergence. Effective Sample Size (ESS) was also examined to confirm sufficient posterior sampling and avoid autocorrelation issues [42]. Model fit was evaluated using the Widely Applicable Information Criterion (WAIC) and Leave-One-Out Cross-Validation Information Criterion (LOOIC), supplemented by cross-validation methods to detect high-influence observations and ensure model stability [43]. As part of the LOO cross-validation process, Pareto k values were examined, confirming that all values remained below 0.70, indicating that parameter estimates were not influenced by high-leverage observations [44].

To ensure sampling stability, posterior distributions and MCMC trace plots were examined for the final selected model to confirm convergence and the absence of trends or irregular fluctuations. The posterior means and 95% HDIs of the fixed-effect coefficients are presented on the log-odds scale. Additionally, mean path estimates were extracted from the posterior distributions, and the 95% HDI was used to quantify the direct and indirect associations between depressive symptoms and inpatient healthcare utilization, providing further insights into the potential role of the mediation mechanism.

### 2.6 Sensitivity analysis

To assess the robustness of the findings, sensitivity analysis was conducted by varying prior distributions and comparing alternative model specifications.

Prior sensitivity analysis was conducted using flat priors for regression coefficients and highly diffuse Student-t (1, 0, 1000) priors for intercepts and ordinal thresholds, to approximate non-informative settings and allow the data to primarily drive posterior estimation.

Model specification sensitivity analysis involved a comparison between a comprehensive model, which included all theoretically relevant variables, and a refined model that retained only variables with stronger posterior evidence. All models were estimated using identical MCMC configurations to ensure comparability. This approach is consistent with [45], who emphasized that Bayesian sensitivity analysis should account for both prior assumptions and structural choices in model formulation.

## 3. Results

Data from 7,777 respondents were analyzed, with a median age of 67 years (IQR 63.0–72.0). Of these, 3,760 (48.35%) were female, and 2,974 (38.24%) reported depressive symptoms. 1,495 participants (19.22%) were hospitalized within the past year. Table 2 provides an overview of participant characteristics.

**Table 2:** Characteristics of participants.

Variables	n (%)
N	7777
**Depressive symptoms**	
No	4803(61.76)
Yes	2974(38.24)
**Inpatient healthcare utilization**	
No	6282(80.78)
Yes	1495(19.22)
**Gender**	
Female	3760(48.35)
Male	4017(51.65)
**Age (year)**	
60-69	5041(64.82)
70-79	2267(29.15)
≥80	469(6.03)
**Marital status**	
No	1399(17.99)
Yes	6378(82.01)
**Ethnicity**	
Non-Han	524(6.74)
Han	7253(93.26)
**Education**	
Illiterate	1996(25.67)
Primary	3612(46.44)
Secondary	1840(23.66)
Higher	329(4.23)
**Residence**	
Urban	2083(26.78)
Rural	5694(73.22)
**Religious belief**	
No	6966(89.57)
Yes	811(10.43)
**Work status**	
No	3450(44.36)
Yes	4327(55.64)
**Health insurance**	
No	195(2.51)
Yes	7582(97.49)
**Pension**	
No	642(8.25)
Yes	7135(91.74)
**Satisfaction with healthcare services**	
Dissatisfied	1306(16.79)
Neutral	3263(41.95)
Satisfied	3208(41.25)
**Physical examination**	
No	3352(43.10)
Yes	4425(56.90)
**Pain**	
No	4783(61.50)
Yes	2994(38.50)
**Chronic disease**	
None	1094(14.07)
Single disease	1687(21.69)
Multiple diseases	4996(64.24)
**Disability**	
No	4449(57.21)
Yes	3328(42.79)
**ADL**	
No limitation	4743(60.99)
Mildly limited	730(9.39)
Moderately limited	905(11.63)
Severely limited	1399(17.99)
**Health status**	
Poor	2239(28.79)
Neutral	3853(49.54)
Good	1685(21.67)
**Satisfaction with health**	
Dissatisfied	2064(26.54)
Neutral	3652(46.96)
Satisfied	2061(26.50)
**Smoking**	
No	5596(71.93)
Yes	2183(28.07)
**Alcohol use**	
No	5697(71.96)
Yes	2081(28.04)

Multicollinearity was assessed prior to model estimation, and no substantial collinearity was detected. Full diagnostic results are provided in S2 Table.

### 3.1 Comprehensive model

#### 3.1.1 Comprehensive model convergence stability and model evaluation.

The comprehensive model demonstrated excellent convergence, with all Rhat values close to 1.00, confirming stable and reliable MCMC sampling. The effective sample sizes (bulk-ESS = 8943.71, tail-ESS = 12912.28) were well above conventional thresholds, ensuring robustness in parameter estimation.

The comprehensive model exhibited stable parameter estimates and generalizability, with WAIC (105551.54) and LOOIC (105551.78) reflecting model consistency. Cross-validation reinforced model robustness, as all Pareto k values remained below 0.7, confirming that parameter estimates were unaffected by high-influence observations.

These metrics support the comprehensive model’s reliability in capturing the association between depressive symptoms and inpatient healthcare utilization.

#### 3.1.2 Associations of depressive symptoms on inpatient healthcare utilization in the comprehensive model.

As shown in Table 3, the direct association between depressive symptoms and inpatient utilization was 0.17 (95% HDI: 0.04, 0.31), accompanied by a total indirect association of 2.43 (95% HDI: 2.10, 2.76). Combined, these yielded a total association of 2.60, with 93.46% attributable to mediation pathways, indicating the predominantly indirect nature of the association.

**Table 3:** Path coefficients for associations depressive symptoms and inpatient healthcare utilization in the comprehensive model.

Variables	Indirect associations via M (X-M-Y)			C-Y Mean (95% HDI)	Direct association (X-Y) Mean (95%HDI)
	X-M Mean (95%HDI)	M-Y Mean (95%HDI)	Total Mean (95%HDI)		
**Mediating variables**					
Pain	−1.18 (−1.28, −1.08)	−0.07 (−0.21, 0.07)	0.09 (−0.08, 0.25)	–	–
Alcohol use	−0.47 (−0.58, −0.37)	−0.37 (−0.53, −0.21)	0.17 (0.09, 0.26)	–	–
Disability	0.70 (0.61, 0.79)	0.14 (0.01, 0.27)	0.10 (0.01, 0.19)	–	–
Smoking	−0.08 (−0.18, 0.02)	−0.45 (−0.61, −0.29)	0.04 (−0.01, 0.09)	–	–
Health status	−1.33 (−1.42, −1.24)	−0.68 (−0.85, −0.52)	0.90 (0.67, 1.13)	–	–
Satisfaction with health	−1.33 (−1.42, −1.23)	−0.27 (−0.41, −0.13)	0.36 (0.18, 0.56)	–	–
ADL	0.99 (0.90, 1.08)	0.22 (0.09, 0.34)	0.21 (0.09, 0.34)	–	–
Chronic disease	0.80 (0.69, 0.90)	0.71 (0.51, 0.91)	0.56 (0.39, 0.74)	–	–
X-M-Y (Total indirect associations)	–	–	2.43 (2.10, 2.76)	–	–
X-Y (Direct association)	–	–	–	–	0.17 (0.04, 0.31)
**Control variables**					
Health insurance	–	–	–	1.22 (0.68, 1.79)	–
Physical examination	–	–	–	0.32 (0.19, 0.45)	–
Marital status	–	–	–	−0.04 (−0.20, 0.13)	–
Work status	–	–	–	−0.30 (−0.44, −0.16)	–
Residence	–	–	–	−0.14 (−0.28, 0.02)	–
Satisfaction with healthcare services	–	–	–	0.02 (−0.10, 0.14)	–
Age	–	–	–	0.35 (0.20, 0.51)	–
Gender	–	–	–	0.45 (0.30, 0.60)	–
Ethnicity	–	–	–	0.02 (−0.22, 0.28)	–
Pension	–	–	–	0.10 (−0.13, 0.33)	–
Education	–	–	–	−0.10 (−0.31, 0.10)	–
Religious belief	–	–	–	−0.12 (−0.32, 0.07)	–

**Fig 3:**
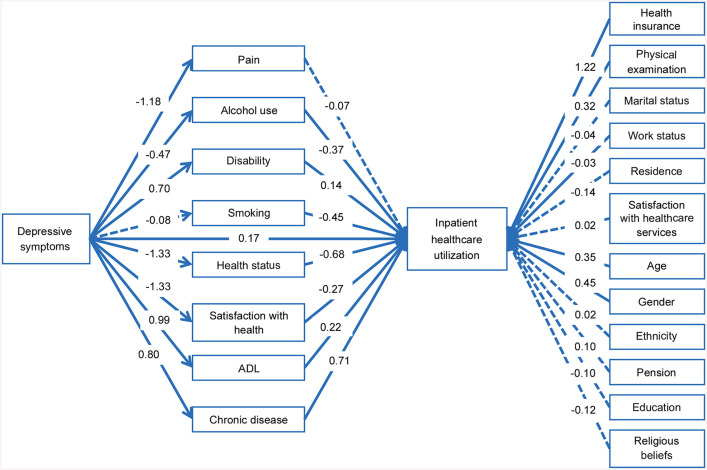
Structural path diagram of the comprehensive model. Note: Solid lines represent associations (log-odds scale) whose 95% HDIs exclude zero. Dashed lines indicate that the 95% HDIs include zero.

Among the eight mediators examined in the comprehensive model, six exhibited posterior estimates with substantial density away from zero, indicating credible support for their mediating roles (Fig 3).

Self-rated health status accounted for the largest indirect association, estimated at 0.90 (95% HDI: 0.67, 1.13). Depressive symptoms were associated with poorer perceived health (−1.33, 95% HDI: −1.42, −1.24), and individuals reporting worse health were more likely to utilize inpatient services (−0.68, 95% HDI: −0.85, −0.52).

The chronic disease pathway showed an indirect association of 0.56 (95% HDI: 0.39, 0.74). Depressive symptoms were positively associated with reporting one or more chronic conditions (0.80, 95% HDI: 0.69, 0.90), which in turn was linked to increased inpatient healthcare utilization (0.71, 95% HDI: 0.51, 0.91).

The pathway involving health satisfaction had an indirect association of 0.36 (95% HDI: 0.18, 0.56). Individuals with depressive symptoms tended to report lower satisfaction with their health (−1.33, 95% HDI: −1.42, −1.23), and lower satisfaction was associated with increased inpatient healthcare utilization (−0.27, 95% HDI: −0.41, −0.13).

For ADL limitations, the model estimated an indirect association of 0.21 (95% HDI: 0.09, 0.34). Depressive symptoms were associated with greater limitation in ADL (0.99, 95% HDI: 0.90, 1.08), which was in turn linked to a higher probability of inpatient service use (0.22, 95% HDI: 0.09, 0.34).

The indirect association through disability was 0.10 (95% HDI: 0.01, 0.19). Depressive symptoms were linked to a higher probability of reporting a disability (0.70, 95% HDI: 0.61, 0.79), and individuals with disabilities were more likely to use inpatient services (0.14, 95% HDI: 0.01, 0.27).

The alcohol use pathway showed an indirect association of 0.17 (95% HDI: 0.09, 0.26). Depressive symptoms were negatively associated with alcohol use (−0.47, 95% HDI: −0.58, −0.37), while alcohol use itself was inversely related to inpatient utilization (−0.37, 95% HDI: −0.53, −0.21), resulting in a positive mediation association.

In contrast, the mediating roles of pain and smoking were less well supported. The path from pain to inpatient healthcare utilization was (−0.07, 95% HDI: −0.21, 0.07), indicating high uncertainty in association direction. Similarly, the path from depressive symptoms to smoking was (−0.08, 95% HDI: −0.18, 0.02), with limited posterior concentration and weak evidence for a stable association.

Several control variables demonstrated substantial associations with inpatient healthcare utilization, with posterior distributions notably concentrated away from zero. Individuals with health insurance showed a higher likelihood of inpatient use (1.22, 95% HDI: 0.68, 1.79), and this pattern was also observed among those who had received a physical examination in the past year (0.32, 95% HDI: 0.19, 0.45). Compared to females, males had a greater probability of using inpatient services (0.45, 95% HDI: 0.30, 0.60). Inpatient utilization also increased across age categories (0.35, 95% HDI: 0.20, 0.51), and was more common among individuals who were not currently working (−0.30, 95% HDI: −0.44, −0.16).

By comparison, marital status, residence, ethnicity, pension, religious belief, satisfaction with healthcare services, and education contributed minimally to the model, as their posterior estimates were close to zero and accompanied by uncertainty intervals.

### 3.2 Refined model

#### 3.2.1 Variable selection for the refined model.

The refined model was specified as an alternative to the comprehensive model, with adjustments made to the variable structure while maintaining alignment with the theoretical framework. The refined model retained variables that exhibited interpretable association patterns in the comprehensive model, including alcohol use, disability, health status, satisfaction with health, ADL limitations, and chronic diseases as mediators. The control variables incorporated in the model were health insurance, physical examination, work status, gender, and age. This configuration aimed to achieve a more parsimonious model structure while preserving theoretical coherence, thereby supporting subsequent comparison and evaluation.

#### 3.2.2 Refined model convergence stability and model evaluation.

Despite its simplified structure, the refined model maintained strong convergence and stability. All Rhat values were close to 1.00, and the effective sample sizes (bulk-ESS = 8180.29; tail-ESS = 12528.92) met standard criteria for reliable parameter estimation. WAIC (86497.73) and LOOIC (86497.79) were closely aligned, indicating strong model fit. All Pareto k values were below 0.7, with no evidence of influential observations affecting estimation.

These results confirm the refined model’s robustness in characterizing the relationship between depressive symptoms and inpatient healthcare utilization.

#### 3.2.3 Associations of depressive symptoms on inpatient healthcare utilization in the refined model.

The refined model produced an association structure that closely mirrored the comprehensive specification, both in magnitude and direction. All estimates were independently re-evaluated based on a reduced set of mediators and control variable, while the structural logic and theoretical framework remained intact. The direct association between depressive symptoms and inpatient utilization was estimated at 0.16 (95% HDI: 0.02, 0.29), while an additional 2.32 (95% HDI: 2.02, 2.62) was transmitted through indirect pathways. Together, these yielded a total association of 2.48, with approximately 93.55% of the association operating through mediators (Table 4).

**Table 4:** Path coefficients for associations depressive symptoms and inpatient healthcare utilization in the refined model.

Variables	Indirect associations via M(X-M-Y)			C-Y Mean (95% HDI)	Direct association (X-Y) Mean (95%HDI)
	X-M Mean (95%HDI)	M-Y Mean (95%HDI)	Total Mean (95%HDI)		
**Mediating variables**					
Alcohol use	−0.48 (−0.59, −0.37)	−0.41 (−0.57, −0.26)	0.20 (0.11, 0.29)	–	–
Disability	0.70 (0.60, 0.79)	0.13 (0.00, 0.26)	0.09 (0.00, 0.18)	–	–
Health Status	−1.33 (−1.42, −1.23)	−0.67 (−0.83, −0.50)	0.89 (0.66, 1.11)	–	–
Satisfaction with health	−1.33 (−1.42, −1.23)	−0.28 (−0.41, −0.14)	0.37 (0.18, 0.54)	–	–
ADL	0.99 (0.89, 1.08)	0.19 (0.07, 0.32)	0.19 (0.07, 0.32)	–	–
Chronic disease	0.80 (0.69, 0.90)	0.74 (0.54, 0.94)	0.59 (0.42, 0.77)	–	–
X-M-Y (Total indirect associations)	–	–	2.32 (2.02, 2.62)	–	–
X-Y (Direct association)	–	–	–	–	0.16 (0.02, 0.29)
**Control variables**					
Health insurance	–	–	–	1.28 (0.78, 1.86)	–
Physical examination		–	–	0.34 (0.21, 0.47)	–
Work status	–	–	–	−0.36 (−0.49, −0.23)	–
Age	–	–	–	0.37 (0.23, 0.52)	–
Gender	–	–	–	0.28 (0.14, 0.40)	–

**Fig 4:**
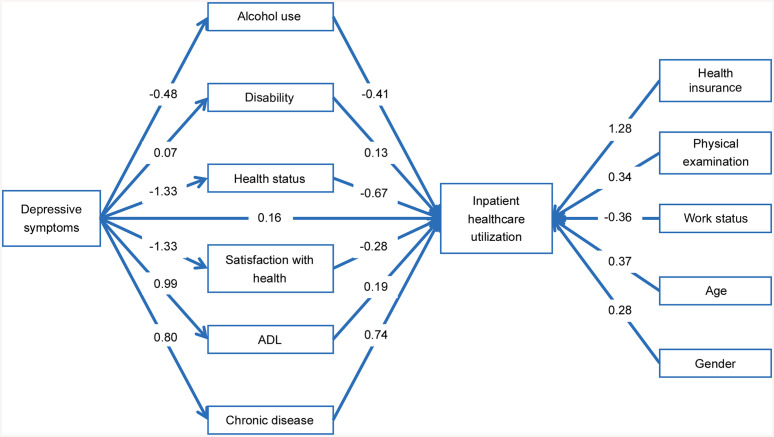
Structural path diagram of the refined model.

Among the retained mediators, health status accounted for the largest portion of the indirect association (0.89, 95% HDI: 0.66, 1.11), followed by chronic disease (0.59, 95% HDI: 0.42, 0.77) and satisfaction with health (0.37, 95% HDI: 0.18, 0.54). Additional contributions were observed for alcohol use (0.20, 95% HDI: 0.11, 0.29), ADL limitations (0.19, 95% HDI: 0.07, 0.32), and disability (0.09, 95% HDI: 0.00, 0.18). The direction and relative ordering of indirect associations remained consistent with those found in the comprehensive model, indicating that the mediating roles of the retained variables followed a similar pattern (Fig 4).

Several control variables also demonstrated credible associations with inpatient healthcare utilization. Individuals with health insurance had the highest estimated likelihood of use (1.28, 95% HDI: 0.78, 1.86), followed by those in higher age groups (0.37, 95% HDI: 0.23, 0.52), those who had received a physical examination in the past year (0.34, 95% HDI: 0.21, 0.47), and male gender (0.28, 95% HDI: 0.14, 0.40). In contrast, work status was inversely associated with inpatient healthcare utilization, with higher use observed among individuals not currently working (−0.36, 95% HDI: −0.49, −0.23). These associations followed the same directional trends as in the comprehensive model.

### 3.3 Model selection

Based on a systematic comparison of convergence diagnostics and estimation reliability, both the comprehensive and refined models demonstrated satisfactory performance. Rhat values were uniformly 1.00, and the effective sample sizes (bulk-ESS and tail-ESS) were sufficient to ensure stable posterior estimation. The two models exhibited comparable generalization capacity (WAIC: 86497.73 vs. 105551.54; LOOIC: 86497.79 vs. 105551.78). Considering its simplified structure, consistent path estimates, and stronger explanatory parsimony, the refined model was selected as the final analytical framework.

**Fig 5:**
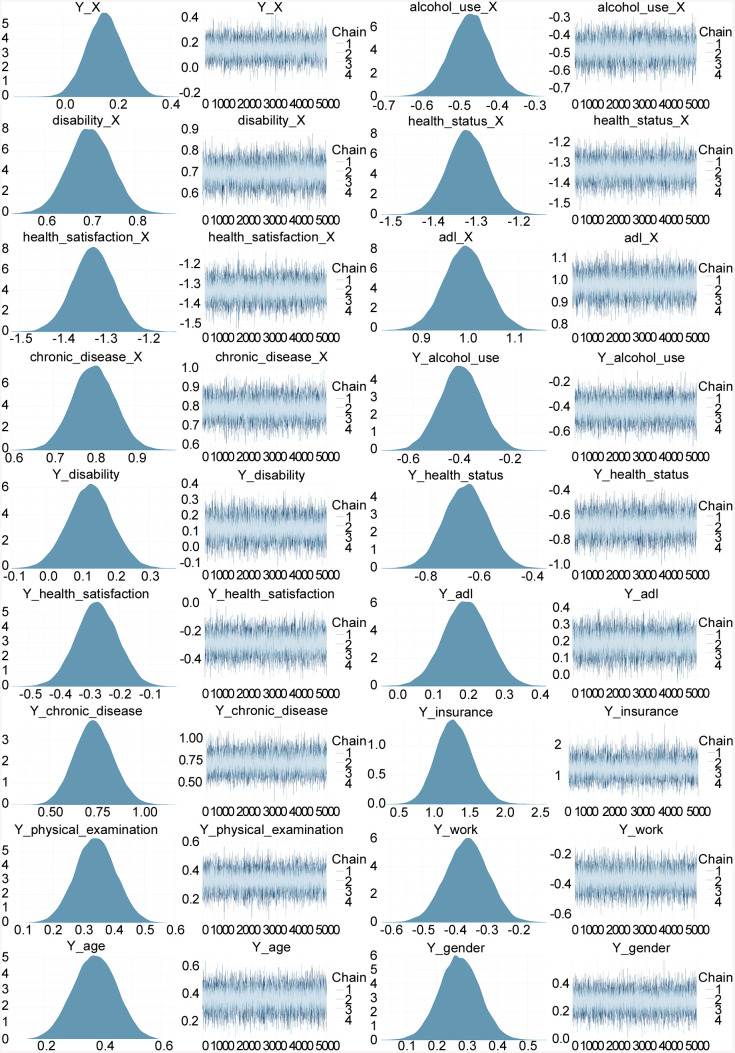
Density and trace plots for the refined model. Note: _X indicates parameters where depressive symptoms are explanatory variables, and Y_ indicates parameters where inpatient healthcare utilization is the outcome.

To further validate the robustness of the refined model, additional evaluation and posterior diagnostics were conducted (Fig 5). MCMC trace plots confirmed well-mixed chains with no discernible trends or irregular fluctuations, supporting estimation stability. The posterior distributions were smooth and bell-shaped, indicating sufficient sampling and reliable parameter estimation.

In addition, Sensitivity analyses showed results consistent with the refined model, with no substantial differences in key associations (see S3 File). All R-hat values were approximately 1.00, and WAIC and LOOIC were nearly identical, further supporting the model’s robustness.

### 3.4 Structural presentation of the integrative framework

**Fig 6:**
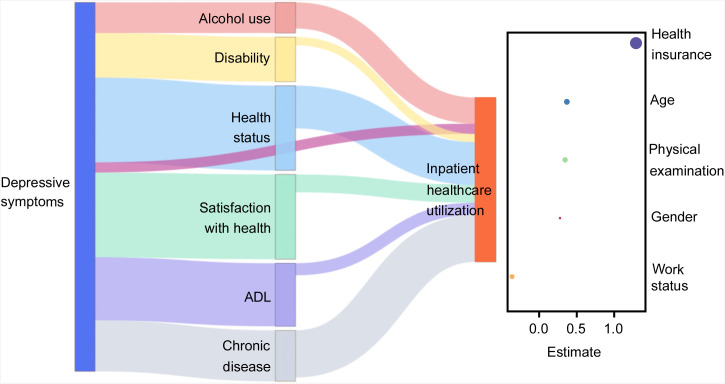
Integrative framework of depressive symptoms and inpatient healthcare utilization Note: Left: mediators and direct paths; Right: control variables. Connection width and dot size reflect posterior means.

The primary analytical output of this study is presented in Fig 6, which illustrates the structural pathways linking depressive symptoms with inpatient healthcare utilization. The framework summarizes both mediated and controlled associations, capturing the combined contributions of six mediating variables (alcohol use, disability, health status, satisfaction with health, ADL and chronic disease) and five control factors (health insurance, age, physical examination, gender and work status). This figure is constructed based on the results of the refined model and serves as the synthesized structural output of the final framework.

## 4. Discussion

Guide by Andsersen behavioral model, this study examined how depressive symptoms are associated with inpatient healthcare utilization among older adults in China. By deconstructing total associations, the model revealed that approximately 93.55% of the association was transmitted through indirect pathways, suggesting a possible dominant role of mediating mechanisms. These findings directly address the study’s aim, indicating that the association of depressive symptoms on inpatient healthcare utilization is predominantly mediated through a system of intervening variables.

The direct association between depressive symptoms on healthcare utilization has been reported in previous CHARLS-based studies [46]. However, these studies primarily focused on overall associations without investigating the underlying mechanisms contributing to this relationship. Building on this empirical foundation, the present study extends prior research by identifying and quantifying the specific mediating pathways through which depressive symptoms may influence inpatient healthcare utilization, thereby advancing both theoretical and analytical understanding in this field.

Our results show that depressive symptoms were associated with inpatient healthcare utilization through multiple mediating pathways, with self-rated health status appearing as the strongest. Individuals experiencing depressive symptoms tended to report lower self-ratings of their health status [47], which may partly account for the increased likelihood of hospitalization. Prior studies have shown that older adults with poorer self-rated health are more likely to access healthcare services, including community-based care [48], highlighting the potential importance of subjective health perception in shaping healthcare-seeking behavior.

Chronic disease ranked second among the mediating pathways in this analysis. Depressive symptoms were associated with a higher likelihood of having multiple chronic diseases, which may be linked to behavioral disruption, access-related, and physiological mechanisms such as inflammation and hormonal dysregulation [49]. These accumulated conditions, in turn, were associated with increased inpatient service use [50]. This pathway illustrates a potential way in which depressive symptoms may be related to hospitalization through chronic disease burden, highlighting the importance of integrated mental and physical healthcare, particularly within hospital-centered systems.

Satisfaction with health, an affective appraisal of overall well-being, was identified as the third mediating pathway. Although direct evidence linking it to inpatient utilization is limited, prior research suggests that depressive symptoms are associated with lower subjective health satisfaction and diminished internal health evaluations [51]. The present findings support its mediating role and underscore the importance of affective health perceptions in inpatient healthcare utilization among older adults.

Distinct from conventional health-related pathways, the alcohol use mechanism showed a counterintuitive pattern in which individuals with depressive symptoms were less likely to drink while non-drinkers showed a higher likelihood of hospitalization. This finding aligns with Castellanos-Perilla et al. who reported lower alcohol consumption among older adults with depression [52], suggesting that depressive symptoms may be associated with reduced alcohol use. Reduced alcohol consumption among older adults may reflect underlying physical decline, adverse interactions with medications, functional impairment, or fewer opportunities for social engagement, rather than protective health behavior [53].

Finally, although the pathways through ADL limitations and disability demonstrated relatively smaller contributions, our model supported their roles as meaningful mediators. In our study, individuals with depressive symptoms and functional impairments showed higher inpatient utilization. Poorer ADL performance among older adults with depression has previously been reported in China [54] however the relationship between functional limitations and healthcare use has received mixed reports. Mai et al. reported that access to care [55] is often limited for people with ADL dependencies [54]. The observed increase in hospitalization in this study among individuals with ADL limitations and disability may reflect unmet care needs, however this assumption needs further exploration.

Overall, depressive symptoms are indirectly associated with inpatient healthcare utilization through health status, chronic disease, satisfaction with health, alcohol use, ADL limitations, and disability. These factors collectively constitute a well-structured and hierarchically differentiated mechanism network.

Beyond the mediating pathways, the control variables also demonstrated notable path contributions, with posterior estimates indicating consistent directional associations. Health insurance had the strongest association, with insured individuals more likely to be hospitalized, possibly reflecting improved access to care [56]. Work status was negatively associated with hospitalization, suggesting that employed individuals, likely due to better functional status, may have reduced inpatient needs, consistent with the findings of Hyun and Kan [57]. Age showed a positive association with hospitalization, indicating that even within older adults, advancing age is linked to higher inpatient service use [46].

The path contributions of physical examination and gender were relatively small. The association with physical examination may reflect greater detection of health issues through regular contact with the healthcare system [58], while the gender association may relate to differences in gender roles, health awareness, or service preferences, as also reflected in prior evidence that older women are less likely to be hospitalized than men [46]. Overall, these control variables capture fundamental demographic and structural influences on hospitalization, providing essential contextual information beyond the mediating mechanisms.

In the refined model, variables from all three domains of the Andersen behavioral model were retained, reflecting the continued theoretical relevance of the framework after empirical selection. Among the predisposing factors, age, gender, work status, health status, satisfaction with health and alcohol use were included, while health insurance and physical examination were retained as enabling factors, and chronic disease, ADL limitations and disability represented need factors. Our findings illustrates that the refined model not only aligns with the conceptual foundations of the Andersen behavioral model but also provides a pathway framework between depressive symptoms and inpatient healthcare utilization among older adults in China.

In the comprehensive model, although a wide range of variables from all three domains of the Andersen behavioral model were included, some did not show credible path associations and were therefore excluded from the refined model. This may suggest that certain variables failed to yield robust empirical associations within the current analytical framework, highlighting a potential gap between theoretical constructions and their practical applicability. Prior studies have noted that the influence of Andersen behavioral model variables may vary across populations and service types [19]. Rogers et al. [59] further emphasized that the role of contextual variables in modelling is shaped by the specific implementation setting, and that their pathway associations are not inherently fixed. Our findings add to the body of knowledge by delineating empirically supported pathways that clarify how depressive symptoms influence inpatient healthcare utilization among older adults, thereby advancing a more contextually grounded understanding of this complex relationship.

## 5. Strengths and limitations

This study is the first to systematically investigate both the direct and indirect associations between depressive symptoms on inpatient healthcare utilization among older adults in China, using a nationally representative dataset.

An innovative combination of a BGSEM was employed to jointly model multiple mediating pathways within a unified analytical framework. This approach enables stable and reliable parameter estimation through full posterior inference and presents uncertainty using 95% HDIs, thereby enhancing the robustness and transparency of the findings [60,61]. By integrating this methodological advancement with a theory-driven design, the study provides a novel quantitative perspective for understanding the complex mechanisms linking depressive symptoms to inpatient service use in later life.

However, this study has several limitations that should be acknowledged. First, this study utilized data from CHARLS, a nationally representative cross-sectional survey, which imposes several limitations on the interpretation of causal pathways. The cross-sectional design, coupled with inconsistencies in the measurement time frames of certain variables within the CHARLS dataset, limits the ability to verify temporal ordering among variables and further weakens the interpretability of directional pathways. Therefore, the findings can only reveal statistical associations rather than definitive causal relationships. Additionally, cross-sectional data have limited capacity to capture complex mechanisms that may exist in real-world settings, such as bidirectional associations or multiple mediating pathways, constraining the modeling and interpretation of such causal structures. As such, the findings should be interpreted as structural support for theoretically hypothesized relationships rather than empirical confirmation of causal associations.

Second, key variables such as depressive symptoms, ADL limitations, health status, and satisfaction with health were self-reported and may be subject to subjective bias or recall error. Moreover, a portion of the CHARLS data was completed by proxy respondents, which may have introduced additional variability and affected the accuracy and consistency of certain measures. In addition, individuals with incomplete data on key variables were excluded during data cleaning, which may have introduced potential selection bias. Although normalized survey weights and multiple imputation was applied to restore representativeness and reduce bias, differences between included and excluded participants cannot be fully ruled out.

Third, due to the limited range of variables available in the CHARLS dataset, certain factors that may be associated with inpatient healthcare utilization, such as health beliefs, which are important predisposing factors that may influence care decisions, were not adequately captured and thus could not be included in the current model [25]. At the same time, the Andersen’s behavioral model defines its three conceptual factors in broad terms, without clearly specifying the interrelations or measurement boundaries of constructs within them [17]. This conceptual ambiguity, together with its inherently static structure, limits its ability to represent the complex and dynamic processes underlying healthcare utilization in real-world contexts.

Finally, while healthcare utilization encompasses a wide spectrum of services, this study focused solely on inpatient care as an indicator of service use. This may have contributed to an underestimation of the broader impact of depressive symptoms on overall healthcare utilization inclusive of primary care services.

## 6. Future research

Future research could benefit from the inclusion of longitudinal data and a broader array of healthcare service indicators to assess the stability and applicability of the proposed theoretical pathway model across different time and service types.

Further theoretical refinement of the Andersen’s behavioral model, particularly in specifying its conceptual boundaries and clarifying the dynamic relationships among its components, may strengthen its explanatory utility in studies of healthcare utilization. Building upon the current focus on theory-driven mediation modelling, subsequent studies may consider using these findings to inform intervention development. Specifically, designing interventions around key mediating mechanisms may enhance the identification and management of depressive symptoms among older adults and support more effective healthcare utilization. Furthermore, the observed pathways may help identify why certain subgroups of older adults remain underserved, despite having healthcare needs. Future studies could focus on identifying these vulnerable populations and developing tailored strategies to improve the equity and accessibility of service use.

## 7. Conclusions

Guided by Andersen behavioral model, this study employed a BGSEM approach to examine the pathways through which depressive symptoms may be associated with inpatient healthcare utilization among older adults in China. The results suggest that this relationship may be largely mediated through specific variables, including self-rated health status, chronic disease, ADL limitations, satisfaction with health, disability, and alcohol use, while the direct associations of depressive symptoms appear relatively limited. These results highlight the importance of intermediary mechanisms linking mental health and healthcare behavior and offer a novel and robust empirical foundation for future mechanism-informed strategies to improve healthcare engagement among older persons experiencing depression.

## Supporting information

S1 File Detailed construction and rationale of variables based on the Andersen behavioral model. (DOCX)

S2 File Construction of variables based on the CHARLS study. (DOCX)

S3 File Convergence diagnostics and posterior estimates from sensitivity analysis. (DOCX)

S1 Table Summary of missing data by variables. (DOCX)

S2 Table Adjusted GVIFs for all explanatory variables. (DOCX)
