# Supplementary material for: Depressive symptoms and healthcare utilization among older adults in China: A cross-sectional examination of the national CHARLS data guided by Andersen behavioral model
Source: PLoS One. 2025 Dec 4;20(12):e0337835. doi: 10.1371/journal.pone.0337835 (PMC12677493; doi:10.1371/journal.pone.0337835)
Supplement: S1 File — (DOCX) [file pone.0337835.s001.docx]

**Supplementary File 1. Detailed construction and rationale of variables based on the Andersen behavioral model**

| **Components of Andersen behavioral model** [1] | **Variable selection** | **Rationale of selection** |
| --- | --- | --- |
| **Predisposing:**  Characteristics existing prior to illness that shape an individual’s propensity to seek and use healthcare. | gender, age, marital status, ethnicity, residence, religious belief, work status, smoking, and alcohol use. | According to the Andersen Behavioral Model and informed by two systematic reviews [2,3], ensuring theoretical consistency with the model’s three domains and alignment with available measures in CHARLS. |
| **Enabling:**  Resources and structural conditions that permit or facilitate access to healthcare services. | education level, pension, satisfaction with healthcare services, physical examination, and health insurance |  |
| **Needs:**  Indicators of perceived or professionally evaluated illness that directly determine healthcare use. | pain, chronic disease, disability, ADL, health status, and satisfaction with health. |  |

**References**

1. Andersen R. A behavioral model of families’ use of health services. Research Series No. 25. Chicago (IL): Center for Health Administration Studies, University of Chicago; 1968

2. Babitsch B, Gohl D, von Lengerke T. Re-revisiting Andersen’s behavioral model of health services use: a systematic review of studies from 1998–2011. GMS Psycho-Soc Med. 2012;9:Doc11. doi: 10.3205/psm000089. PMID: 23133505.

3. Alkhawaldeh A, ALBashtawy M, Rayan A, Abdalrahim A, Musa A, Eshah N, et al. Application and use of Andersen’s behavioral model as theoretical framework: a systematic literature review from 2012–2021. Iran J Public Health. 2023;52(7):1437–46. doi: 10.18502/ijph.v52i7.12584. PMID: 37593505.
