# Supplementary material for: Depressive symptoms and healthcare utilization among older adults in China: A cross-sectional examination of the national CHARLS data guided by Andersen behavioral model
Source: PLoS One. 2025 Dec 4;20(12):e0337835. doi: 10.1371/journal.pone.0337835 (PMC12677493; doi:10.1371/journal.pone.0337835)
Supplement: S2 File — (DOCX) [file pone.0337835.s002.docx]

**Supplementary File 2. Construction of variables based on the CHARLS study**

Our study constructs key variables related to health and healthcare utilization among older adults using data from the China Health and Retirement Longitudinal Study (CHARLS). The CHARLS dataset provides a comprehensive national sample of Chinese adults aged 45 and above, with detailed information on demographics, health conditions, healthcare access, and socioeconomic factors.

Below, we provide a detailed description of the variables included in our study. These variables were derived by restructuring, recording, and aggregating relevant survey responses from CHARLS to align with the Andersen behavioral model.

**Independent variables**

**Depressive symptoms**

Definition and coding: Depressive symptoms were assessed using the Center for Epidemiologic Studies Depression Scale (CES-D-10), which consists of 10 items (dc009 to dc018) measuring depressive affect and mood over the past week.

Each item was originally recorded with four response categories:

1. Rarely or None (<1 Day)

2. Some or A Little (1 - 2 Days)

3. Occasionally or Moderate Amount of Time (3 - 4 Days)

4. Most of the Time (5 - 7 Days)

Responses coded as 8 (Don't Know) and 9 (Refused) were treated as missing values.

For analysis, each item was recorded as follows:

1 → 0 (Rarely or None)

2 → 1 (Some or A Little)

3 → 2 (Occasionally or Moderate Amount of Time)

4 → 3 (Most of the Time)

Reverse Scored Items

Two positively worded items (dc013: "Felt hopeful about the future" and dc016: "I was happy") were reverse-scored to align with the depressive symptoms scale:

0 → 3

1 → 2

2 → 1

3 → 0

The final depression variable was computed as the sum of all 10 items (dc009 to dc018), with higher scores indicating more severe depressive symptoms.

For analysis, this variable was recoded into a binary format:

0 = No depressive symptoms (CES-D-10 score <10)

1 = Presence of depressive symptoms (CES-D-10 score ≥10)

The original dc009 to dc018 variables were then combined and renamed as depression in the dataset.

**Dependent variables**

**Inpatient healthcare utilization**

Definition and coding: Inpatient healthcare utilization was recorded under the variable ee003: Received Inpatient Care, which captured whether respondents had been hospitalized in the past year.

The original responses were coded as follows:

1. Yes (Received inpatient care)

2. No (Did not receive inpatient care)

For analysis, this variable was recoded into a binary format:

1 = Used inpatient services (Category 1)

0 = Did not use inpatient services (Category 2)

The original variable ee003 was then renamed as inpatient in the dataset.

**Variable definitions and coding based on the Andersen behavioral model**

**Predisposing factors**

**Gender**

Definition and coding: Gender was recorded under the variable bg002: Gender, which classified respondents as male or female.

For analysis, this variable was recoded into a binary format:

0 = Female

1 = Male

The original variable bg002 was then renamed as gender in the dataset.

**Age**

Definition and coding: Age was derived from the respondents’ year of birth recorded in the dataset. The year of birth was subtracted from the survey year (2018) to calculate the exact age. Participants aged below 60 were excluded from the analysis. The remaining individuals were categorized into three age groups:

0 = 60 - 69 years

1 = 70 - 79 years

2 = ≥80 years

Age was treated as a categorical variable to improve model convergence and to ensure consistent and stable interpretation of results within the Bayesian generalized structural equation modeling framework.

**Marital status**

Definition and coding: Marital status was recorded under the variable bd003: Marital Status, with six categories:

1. Married with spouse present

2. Married but not living with a spouse temporarily (e.g., due to work)

3. Separated

4. Divorced

5. Widowed

6. Never married

For analysis, this variable was recoded into a binary format:

0 = Not married (Categories 3, 4, 5, and 6)

1 = Currently married (Categories 1 and 2)

The original variable bd003 was then renamed as marital in the dataset.

**Ethnicity**

Definition and coding: Ethnicity was recorded under the variable bg001_w4: Nation, with 11 categories:

1. Han nationality

2. Zhuang nationality

3. Manchu nationality

4. Hui nationality

5. Miao nationality

6. Uyghur nationality

7. Tujia nationality

8. Yi nationality

9. Mongol nationality

10. Tibetan nationality

11. Other ethnic groups

For analysis, this variable was recoded into a binary format:

0 = Non-Han nationality (Categories 2 - 11)

1 = Han nationality (Category 1)

The original variable bg001_w4 was then renamed as nation in the dataset.

**Residence**

Definition and coding: Residential location was recorded under the variable bb001_w3_2: Location of Residential Address, with four categories:

1. Central of city/town

2. Urban-rural integration zone

3. Rural

4. Special zone

For analysis, individuals in the "Special Zone" (Category 4) were excluded. The remaining respondents were recorded into a binary format:

0 = Urban (Categories 1 and 2)

1 = Rural (Category 3)

The original variable bb001_w3_2 was then renamed as residential in the dataset.

**Religious belief**

Definition and coding: Religious belief was recorded under the variable bg004: Religious Belief as a binary variable:

0 = No religious belief

1 = Has religious belief

The original variable bg004 was then renamed as religious in the dataset.

**Work status**

Definition and coding: Working status was recorded under the variable xf1: Ever Worked but Currently Not Working, with three categories:

1. Currently not working

2. Never worked

3. Currently working

For analysis, this variable was recoded into a binary format:

0 = Not working (Categories 1 and 2)

1 = Currently working (Category 3)

The original variable xf1 was then renamed as work in the dataset.

**Smoking**

Definition and coding: Smoking status was derived from multiple variables in the dataset, including:

zsmoke: Ever Smoked at ZIW Time (1 = Yes)

da061_w4: Still Smoke or Already Quit (1 = Still Smoking, 2 = Quit, 3 = Never Smoked)

da059: Ever Smoked (1 = Yes, 2 = No)

da061: Still Smoke or Already Quit (1 = Still Smoking, 2 = Quit)

For analysis, this variable was recoded into a binary format:

0 = Not smoking (including never smoked or quit smoking) (Categories 2 and 3 from da061_w4 or category 2 from da061)

1 = Currently smoking (Category 1 from da061_w4 or da061)

The original smoking-related variables were then combined and renamed as smoke in the dataset.

**Alcohol use**

Definition and coding: Alcohol consumption was recorded under the variable da067: Frequency of Drinking Alcoholic Beverages in the Past Year, with three categories:

1. Drinks more than once a month

2. Drinks but less than once a month

3. Does not drink

For analysis, this variable was recoded into a binary format:

1 = Drinker (Category 1)

0 = Non-drinker (Categories 2 and 3)

The original variable da067 was then renamed as alcohol in the dataset.

**Enabling factors**

**Education**

Definition and coding: Educational attainment was recorded under the variable bd001_w2_4: Education, with 11 categories:

1. No formal education (Illiterate)

2. Did not finish primary school / Sishu / Home school

3. Primary school

4. Middle school

5. High school

6. Vocational school

7. Two-/Three-year college / Associate degree

8. Four-year college / Bachelor’s degree

9. Master’s degree

10. Doctoral degree / Ph.D.

For analysis, this variable was recorded into four groups:

0 = Illiterate (Category 1)

1 = Primary education (Categories 2 and 3)

2 = Secondary education (Categories 4, 5, and 6)

3 = Higher education (Categories 7, 8, 9, and 10)

The original variable bd001_w2_4 was then renamed as education in the dataset.

**Pension**

Definition and coding: Pension status was recorded using multiple variables in the dataset, capturing different types of pension participation and receipt. The original dataset included the following variables:

fn002_w4: Government/Institution/Firm Pension

fn030_w4: Supplemental Pension

fn058_w4: Residents/New Rural/Urban Residents Pension

fn103_w4: Old Rural Pension

fn073_w4: Life Insurance Pension

fn079_w2_3: Land Expropriation Pension Insurance

fn043_w4: Commercial Pension

fn083_w2: Other Pension Programs

For analysis, these variables were recorded into binary indicators:

For fn002_w4, fn030_w4, fn058_w4, fn103_w4, fn073_w4:

1 = Yes (Participated in the pension program)

2 = No (Did not participate) → Recoded as 0

For fn079_w2_3, fn043_w4, fn083_w2:

1 = Yes, but not receiving & 2 = Yes, receiving → Recoded as 1

3 = No participation → Recoded as 0

The final pension variable was computed by summing these binary indicators, and individuals were classified as follows:

0 = No pension (No participation in any pension scheme)

1 = Has pension (Participating in at least one type of pension scheme)

The original pension-related variables were then combined and renamed as pension in the dataset.

**Satisfaction with healthcare services**

Definition and coding: Satisfaction with healthcare services was recorded under the variable eh007_w3: Satisfied with Local Healthcare Services, with five categories:

1. Very satisfied

2. Somewhat satisfied

3. Neutral

4. Somewhat dissatisfied

5. Very dissatisfied

For analysis, this variable was recorded into a three-category format:

0 = Dissatisfied (Categories 4 and 5)

1 = Neutral (Category 3)

2 = Satisfied (Categories 1 and 2)

The original variable eh007_w3 was then renamed as satisfiedservice in the dataset.

**Physical examination**

Definition and coding: Physical examination was recorded using two variables:

ec001: Time of Taking Last Physical Examination (for new respondents)

ec001_w4: Time of Last Physical Examination (for follow-up respondents)

To ensure consistency, responses were merged into a single variable, prioritizing available responses:

If ec001 was available, its value was used

If ec001 was missing, ec001_w4 was used instead

Both ec001 and ec001_w4 contained the same response categories:

1. Had a physical examination (Year and Month recorded)

2. Never had a physical examination

For analysis, this variable was recoded into a binary format:

0 = Never had a physical examination (Category 2)

1 = Had a physical examination (Category 1)

The original variables ec001 and ec001_w4 were then combined and renamed as examination in the dataset.

**Health insurance**

Definition and coding: Insurance status was recorded under the variable ea001_w4_s12: No Insurance, with two categories:

0. Has insurance

12. No insurance

For analysis, this variable was recoded into a binary format:

0 = No insurance (Category 12)

1 = Has insurance (Category 0)

The original variable ea001_w4_s12 was then renamed as insurance in the dataset.

**Need factors**

**Pain**

Definition and coding: Self-reported bodily pain was recorded under the variable da041_w4: Troubled with Body Pain, with five categories:

1. None

2. A little

3. Somewhat

4. Quite a bit

5. Very

For analysis, this variable was recoded into a binary format:

1 = No pain (Category 1)

0 = Experienced pain (Categories 2, 3, 4, and 5)

The original variable da041_w4 was then renamed as pain in the dataset.

**Chronic disease**

Definition and coding: Chronic disease status was constructed using two sources:

zdiagnosed_*: Doctor-diagnosed chronic diseases recorded in the previous survey wave.

da007_*: Self-reported doctor-diagnosed chronic diseases recorded in the current survey wave.

The dataset included 14 chronic diseases, which were originally recorded as separate binary variables:

1.Hypertension

2. Dyslipidemia

3. Diabetes

4. Cancer

5. Chronic lung diseases

6. Liver disease

7. Heart attack

8. Stroke

9. Kidney disease

10. Stomach disease

11. Emotional problems

12. Memory-related diseases

13. Arthritis

14. Asthma

For analysis, the zdiagnosed_* and da007_* variables were merged, where zdiagnosed_* values were used when available; otherwise, da007_* values were used.

Each individual chronic disease variable was originally coded as:

1.Diagnosed with the disease

2. Not diagnosed (Recoded as 0 in analysis)

The final chronic disease variable was computed as the sum of all diagnosed conditions and was recorded into three categories:

0 = No chronic disease (sum = 0)

1 = One chronic disease (sum = 1)

2 = Two or more chronic diseases (sum ≥2)

The original chronic disease variables zdiagnosed_ and da007_ were then combined and renamed as disease in the dataset.

**Disability**

Definition and coding: Disability status was constructed using two sources:

zdisability_*: Doctor-diagnosed disabilities recorded in the previous survey wave.

da005_*: Self-reported doctor-diagnosed disabilities recorded in the current survey wave.

The dataset included five types of disabilities, which were originally recorded as separate binary variables:

1. Physical disabilities

2. Brain damage or mental retardation

3. Vision problems

4. Hearing problems

5. Speech impediments

For analysis, the zdisability_* and da005_* variables were merged, where zdisability_* values were used when available; otherwise, da005_* values were used.

Each individual disability variable was originally coded as:

1. Diagnosed with the disability

2. Not diagnosed (Recoded as 0 in analysis)

The final disability variable was computed as the sum of all diagnosed disabilities and was recorded into a binary format:

0 = No disability (sum = 0)

1 = At least one disability (sum ≥1)

The original disability-related variables zdisability_ and da005_ were then combined and renamed as disability in the dataset.

**ADL (Activities of Daily Living) limitations**

Definition and coding: ADL limitations were constructed using two sets of variables:

Basic Activities of Daily Living (BADL):

db010: Dressing

db011: Bathing or showering

db012: Eating

db013: Getting into or out of bed

db014: Using the toilet

db015: Controlling urination and defecation

Instrumental Activities of Daily Living (IADL):

db016: Household chores

db017: Preparing hot meals

db018: Shopping for groceries

db019: Managing money

db020: Taking medications

Each BADL and IADL variable was originally coded as follows:

1. No difficulty

2. Has difficulty but can still do it

3. Has difficulty and needs help

4. Cannot do it

For analysis, these variables were recorded as binary indicators:

0 = No limitation (originally coded as 1)

1 = Any level of difficulty (originally coded as 2, 3, or 4)

The total number of limitations in BADL and IADL were then computed, and the final ADL variable was categorized as follows:

0 = No limitation (BADL = 0 and IADL = 0)

1 = Mild limitation (BADL ≥ 1 and IADL = 0)

2 = Moderate limitation (BADL ≥ 1 and difficulty in at least one of db017, db018, or db020)

3 = Severe limitation (BADL ≥ 1 or IADL ≥ 1)

The original BADL and IADL variables db010–db020 were then combined and renamed as adl in the dataset. This ADL scoring and classification approach was adapted from the ADL Scoring Method based on Gong et al. [1].

**Health status**

Definition and coding: Self-reported health status was recorded under the variable da002: Self-Reported Health Status, with five categories:

1. Very good

2. Good

3. Fair

4. Poor

5. Very poor

For analysis, this variable was recorded into a three-category format:

2 = Good health (Categories 1 and 2)

1 = Neutral health (Category 3)

0 = Poor health (Categories 4 and 5)

The original variable da002 was then renamed as health status in the dataset.

**Satisfaction with health**

Definition and coding: Self-reported health satisfaction was recorded under the variable dc042_w3: Health Satisfaction, with five categories:

1. Completely satisfied

2. Very satisfied

3. Somewhat satisfied

4. Not very satisfied

5. Not at all satisfied

For analysis, this variable was recorded into a three-category format:

2 = Satisfied (Categories 1 and 2)

1 = Neutral (Category 3)

0 = Dissatisfied (Categories 4 and 5)

The original variable dc042_w3 was then renamed as health_satisfaction in the dataset.

**References**

1. Gong J, Wang G, Wang Y, Chen X, Chen Y, Meng Q, et al. Nowcasting and forecasting the care needs of the older population in China: analysis of data from the China Health and Retirement Longitudinal Study (CHARLS). Lancet Public Health. 2022;7(12):e1005–13. doi: 10.1016/S2468-2667(22)00221-8. PMID: 36423656.
