# Supplementary material for: Depressive symptoms and healthcare utilization among older adults in China: A cross-sectional examination of the national CHARLS data guided by Andersen behavioral model
Source: PLoS One. 2025 Dec 4;20(12):e0337835. doi: 10.1371/journal.pone.0337835 (PMC12677493; doi:10.1371/journal.pone.0337835)
Supplement: S3 File — (DOCX) [file pone.0337835.s003.docx]

**Supplementary File 3. Convergence diagnostics and posterior estimates from sensitivity analysis**

The sensitivity model, estimated under weakly informative priors (flat priors for regression coefficients and Student-t (1, 0, 1000) priors for intercepts and ordinal thresholds), also demonstrated strong convergence and estimation stability. The sensitivity model was estimated under weakly informative priors, including flat priors for regression coefficients and Student-t (1, 0, 1000) priors for intercepts and ordinal thresholds. All Rhat values were approximately 1.00. The bulk-ESS was 7800.37, and the tail ESS was 13302.70. WAIC was 86497.28 and LOOIC was 86497.29. All Pareto k values were below 0.7.

These results confirm that the posterior inferences are stable and robust to prior assumptions. Posterior means and 95% HDIs for all major paths are reported below.

Effects of Depressive symptoms on inpatient healthcare utilization in the sensitivity analysis model

| **Variables** | **Indirect effects (X-M-Y)** | | | **C-Y**  **Mean**  **(95% HDI)** | **Direct effect (X-Y)**  **Mean (95%HDI)** |
| --- | --- | --- | --- | --- | --- |
|  | **X-M**  **Mean (95%HDI)** | **M-Y**  **Mean (95%HDI)** | **Total**  **Mean**  **(95%HDI)** |  |  |
| **Mediating variables** |  |  |  |  |  |
| Alcohol use | -0.48  (-0.59, -0.37) | -0.42  (-0.58, -0.27) | 0.20  (0.11, 0.29) | - | - |
| Disability | 0.70  (0.61, 0.79) | 0.13  (0.00, 0.25) | 0.09  (0.00, 0.18) | - | - |
| Health status | -1.33  (-1.42, -1.24) | -0.67  (-0.83, -0.51) | 0.89  (0.66, 1.12) | - | - |
| Satisfaction with health | -1.33  (-1.42, -1.23) | -0.28  (-0.41, -0.14) | 0.37  (0.18, 0.54) | - | - |
| ADL | 0.99  (0.90, 1.08) | 0.20  (0.07, 0.31) | 0.19  (0.07, 0.31) | - | - |
| Chronic disease | 0.80  (0.70, 0.90) | 0.74  (0.54, 0.95) | 0.59  (0.41, 0.77) | - | - |
| X-M-Y  (Total indirect effects) | - | - | 2.33  (2.02, 2.62) | - | - |
| X-Y (Direct effect) | - | - | - | - | 0.15  (0.02, 0.29) |
| **Control variables** |  |  |  |  |  |
| Health insurance | - | - | - | 1.31  (0.76, 1.85) | - |
| Physical examination |  | - | - | 0.34  (0.21, 0.47) | - |
| Work status | - | - | - | -0.36  (-0.49, -0.23) | - |
| Age | - | - | - | 0.37  (0.22, 0.52) | - |
| Gender | - | - | - | 0.28  (0.15, 0.41) | - |
