# Supplementary material for: Depressive symptoms and healthcare utilization among older adults in China: A cross-sectional examination of the national CHARLS data guided by Andersen behavioral model
Source: PLoS One. 2025 Dec 4;20(12):e0337835. doi: 10.1371/journal.pone.0337835 (PMC12677493; doi:10.1371/journal.pone.0337835)
Supplement: S1 Table — (DOCX) [file pone.0337835.s004.docx]

**Supplementary Table 1. Summary of missing data by variables**

| **Variables** | **Observed (n)** | **Total (N)** | **Missing (n)** | **Missing (%)** |
| --- | --- | --- | --- | --- |
| **Independent variable** |  |  |  |  |
| Depressive symptoms | 7,777 | 7,777 | 0 | 0.00% |
| **Dependent variable** |  |  |  |  |
| Inpatient healthcare utilization | 7,777 | 7,777 | 0 | 0.00% |
| **Mediating variables** |  |  |  |  |
| Pain | 7,770 | 7,777 | 7 | 0.09% |
| Chronic disease | 7,777 | 7,777 | 0 | 0.00% |
| Disability | 7,777 | 7,777 | 0 | 0.00% |
| ADL | 5,979 | 7,777 | 1,768 | 22.73% |
| Health status | 7,770 | 7,777 | 7 | 0.09% |
| Satisfaction with health | 7,777 | 7,777 | 0 | 0.00% |
| Smoking | 7,769 | 7,777 | 8 | 0.10% |
| Alcohol use | 7,769 | 7,777 | 8 | 0.10% |
| **Control variables** |  |  |  |  |
| Gender | 7,777 | 7,777 | 0 | 0.00% |
| Age | 7,777 | 7,777 | 0 | 0.00% |
| Marital status | 7,777 | 7,777 | 0 | 0.00% |
| Ethnicity | 7,777 | 7,777 | 0 | 0.00% |
| Residence | 7,777 | 7,777 | 0 | 0.00% |
| Education | 7,777 | 7,777 | 0 | 0.00% |
| Pension | 7,770 | 7,777 | 7 | 0.09% |
| Satisfaction with healthcare services | 7,577 | 7,777 | 200 | 2.57% |
| Religious belief | 7,777 | 7,777 | 0 | 0.00% |
| Work status | 7,770 | 7,777 | 7 | 0.09% |
| Health insurance | 7,777 | 7,777 | 0 | 0.00% |
| Physical examination | 7,777 | 7,777 | 0 | 0.00% |
