# Supplementary material for: Depressive symptoms and healthcare utilization among older adults in China: A cross-sectional examination of the national CHARLS data guided by Andersen behavioral model
Source: PLoS One. 2025 Dec 4;20(12):e0337835. doi: 10.1371/journal.pone.0337835 (PMC12677493; doi:10.1371/journal.pone.0337835)
Supplement: S2 Table — (DOCX) [file pone.0337835.s005.docx]

**Supplementary Table 2. Adjusted GVIFs for all explanatory variables**

| **Variables** | **Df** | **GVIF^(1/(2*Df))** |
| --- | --- | --- |
| **Independent variable** |  |  |
| Depressive symptoms | 1 | 1.13 |
| **Mediating variables** |  |  |
| Pain | 1 | 1.12 |
| Chronic disease | 1 | 1.12 |
| Disability | 1 | 1.06 |
| ADL | 3 | 1.05 |
| Health status | 2 | 1.14 |
| Satisfaction with health | 1 | 1.22 |
| Smoking | 1 | 1.15 |
| Alcohol use | 1 | 1.11 |
| **Control variables** |  |  |
| Gender | 1 | 1.30 |
| Age | 1 | 1.09 |
| Marital status | 1 | 1.06 |
| Ethnicity | 1 | 1.01 |
| Residence | 1 | 1.17 |
| Education | 1 | 1.17 |
| Pension | 1 | 1.03 |
| Satisfaction with healthcare services | 2 | 1.02 |
| Religious belief | 1 | 1.02 |
| Work status | 1 | 1.18 |
| Health insurance | 1 | 1.02 |
| Physical examination | 1 | 1.03 |
